# Possible Triggering Effect of Influenza Vaccination on Psoriasis

**DOI:** 10.1155/2015/258430

**Published:** 2015-08-25

**Authors:** Ali Tahsin Gunes, Emel Fetil, Sevgi Akarsu, Ozlem Ozbagcivan, Lale Babayeva

**Affiliations:** Department of Dermatology, Faculty of Medicine, Dokuz Eylul University, Inciraltı, 35340 Izmir, Turkey

## Abstract

Psoriasis is a chronic, recurrent, immune-mediated inflammatory disease and it can be provoked or exacerbated by a variety of different environmental factors, particularly infections and drugs. In addition, a possible association between vaccination and the new onset and/or exacerbation of psoriasis has been reported by a number of different authors. The aim of this study is to investigate the effects of influenza vaccination on patients with psoriasis. Here, we report the findings from 43 patients suffering from psoriasis (clinical phenotypes as mixed guttate/plaque lesions, palmoplantar or scalp psoriasis) whose diseases had been triggered after influenza vaccination applied in the 2009-2010 season. The short time intervals between vaccination and psoriasis flares in our patients and the lack of other possible triggers suggest that influenza vaccinations may have provocative effects on psoriasis. However, further large and controlled studies need to be carried out to confirm this relationship.

## 1. Introduction

Vaccination is a proven and well-established strategy for the prevention of infectious diseases in the general population and in patients with immune-mediated chronic inflammatory diseases. This group of patients has an increased risk of contracting complications of some vaccine-preventable infections due to the nature of the disease and immunomodulatory treatments [1]. However, concerns have emerged regarding the safety of vaccinations in immune-mediated inflammatory diseases following recent publications that highlight a stimulating effect of vaccination at the onset of inflammatory disease or during its course. Although a direct and causal relationship between vaccination and the flare of the disease has not been detected by any substantial research studies, these publications have given rise to a belief among some clinicians that vaccination may have a triggering effect [1–4].

Psoriasisis one of the world's most frequent chronic, recurrent, and inflammatory diseases, affecting around 2% of the population and is characterized by erythematous scaly plaques on the skin [5]. It is now recognized as an immune-mediated inflammatory disease [6]. Furthermore, the principle pathogenetic factor is defined as a T-cell mediated autoimmunity which is directed against poorly defined antigens [7]. On a genetic basis, various endogenous and exogenous triggering factors move the patient from a state of latency to clinical disease. Physical or chemical factors, infections, and various types of medications are the most important among these and they may affect the course of the psoriasis by many different mechanisms [8, 9]. However, induction or worsening of the psoriasis followed by some vaccinations has only rarely been reported in literature. Most of the available publications refer to case reports and a few observational studies [10–17]. Here, we report on the findings from 43 psoriasis patients whose diseases were triggered after influenza vaccination with no other detected possible provoking factors.

## 2. Materials and Methods 

We collected cases that were on the onset of contracting psoriasis or where the disease had worsened, within 3 months following immunization with commercial influenza vaccines that were used in the 2009-2010 season. Trade names and the date of the applications were noted for all patients together with the historical and clinical features of the eruption. The diagnosis of psoriasis and its clinical phenotype classification were established for all patients after clinical and histopathological evaluations by an experienced dermatologist. Patients were asked whether they had used any other drug and/or vaccination prior to their eruption. Also, various factors including focal infections that may have a triggering effect on their psoriasis were investigated. Routine hematological and biochemical analysis, urinalysis, HIV and VDRL tests, throat and urine cultures, Water's and thorax radiographs, and tooth examinations were performed. Patients were enrolled into our study group if no other trigger of psoriasis had been identified such as infection or intake of drugs.

## 3. Results and Discussion

Our observational clinical studyevaluated 43 psoriasis patients whose diseases had been triggered after receiving vaccinations for influenza. Patients had a history of using one of the two types of inactivated influenza vaccine trivalent types A and B (split virion). Among these, Vaxigrip Sanofi Pasteur, which contained three different strains of the influenza virus A/Brisbane/59/2007- (H1N1-) like strain, A/Brisbane/10/2007- (H3N2-) like strain, and B/Brisbane/60/2008-like strain with some adjuvants (e.g., ovalbumin, thimerosal, formaldehyde, and neomycin), was used in 34 of the patients (79.1% of the total sample); Fluarix GlaxoSmithKline Biologicals, which contained three different strains of the influenza virus A/Brisbane/59/2007, IVR-148 (H1N1), an A/Brisbane/10/2007-like virus A (H3N2), and B/Brisbane/60/2008 with some adjuvants (e.g., ovalbumin, formaldehyde, and gentamicin sulfate), were used in 9 of the patients (20.9% of the total sample).

Of the 43 patients (26 female, 17 male), 37 (86%) had mixed plaque type and guttate psoriasis, three of the patients (7%) suffered from palmoplantar psoriasis, and another three (7%) suffered from psoriasis on the scalp. There was an exacerbation of preexisting psoriasis after vaccination in 36 (83.7%) of them while it was the first induction of psoriasis in the remaining 7 (16.3%) patients. The latent period for the induction or exacerbation of psoriasis after vaccination was between 2 weeks and 2 months, but most patients contracted it within a period of 2 to 3 weeks. While 38 (88.4%) patients had a history of vaccination prior to their psoriasis without any other drug intake, the remaining five (11.6% of the sample) patients had a history of using some drugs but these were not identified as being responsible for the induction or exacerbation of psoriasis. The patients in our study group had no other vaccination experience in the past except the influenza vaccine and had no other triggering factors such as infections at the time they were enrolled into the study.

Although increased susceptibility to infection in patients with psoriasis remains a matter of debate, it should be emphasized that some special consideration should be given to vaccination strategies in psoriasis patients, especially in the current era of biological therapies [18]. Despite this, it was reported that the rates of vaccination among patients with psoriasis remain low [19, 20]. Recently, in the largest study conducted by Sbidian et al. to assess the coverage of 2009 monovalent H1N1 influenza vaccination, the overall influenza vaccine coverage was found to be 19% among 1308 French patients with psoriasis. In this study, the patients who had not received vaccination for the H1N1 influenza expressed the risk of vaccine-related adverse effects (54%), uncertainty about the vaccination efficacy (50%), and fear concerning the psoriasis flare triggering effect (17%) as major concerns [19].

Substantial advances have been achieved in understanding the genetics and pathomechanisms of psoriasis in recent years. It is considered to be a primarily Th1-type disease characterized by Th1 cytokines and a predominance of CD8+ cells in the epidermis and CD4+ cells in the dermis [5, 6]. Currently, it is recognized as one of the immune-mediated inflammatory diseases [5]. Increasingly, psoriasis is being understood as an autoimmune disease although no definitive autoantigen or immunogen has been identified responsible for the inflammation [6, 7]. Autoimmunity results from complex interactions between genetic predisposition and environmental factors and can be triggered by a number of stimuli, including local inflammation as well as viral, bacterial, and parasitic infections [3, 4]. Vaccinations may trigger autoimmunity by two mechanisms which are antigen specific or antigen nonspecific as in natural infections [1–4]. Because the vaccines also contain adjuvant materials (e.g., aluminum salts, thiomersal, squalene, sorbitol, albumin, neomycin, and gentamycin), we cannot identify, with certainty, the responsible agent for the autoimmune phenomena either the infectious component of the vaccine or the adjuvant [2, 3, 21]. Most of the reported psoriasis exacerbations in the literature were related with influenza vaccines with adjuvants, although only one patient with guttate psoriasis described an exacerbation after 2009 monovalent H1N1 vaccine infusion without adjuvant [17].

Over the years numerous reports have raised the suspicion of the safety of vaccines in autoimmunity and in persons already diagnosed with autoimmune conditions. Because of the popularity and the widespread use of influenza vaccine, its effects have been examined in many autoimmune conditions [2]. Influenza vaccines contain formol-inactivated, purified influenza virus antigens, with or without adjuvants. The vaccine strain composition is reconsidered each year by the WHO and the European Union. Although each vaccine can have a unique potential to provoke immune-mediated problems, trials comparing the adverse effects of the two types of influenza vaccines (split virion and surface antigen) did not find any significant difference [22]. Although H1N1 vaccines and other seasonal vaccines are generally safe and effective, many serious and nonserious vaccine-related adverse events have been reported [20–23]. The vaccination against influenza has been associated with several autoimmune adverse events including Guillain-Barre syndrome, microscopic polyangiitis, rheumatoid vasculitis, thrombotic thrombocytopenic purpura, and increased antiphospholipid antibodies [3, 24]. Occasionally, some cutaneous adverse effects can also occur at the site of injection or at a distant area as local or generalized reactions. Local reactions including transient redness, induration, edema, pain, and ecchymoses are not rare and are usually related to a nonspecific stimulation of host immune system [22]. Additionally, there have been many reports displaying various cutaneous side effects including a wide range of dysimmune reactions and autoimmune phenomena as well as full-blown autoimmune diseases such as Stevens-Johnson syndrome, urticaria, eczematoid lesions, pityriasis rosea, pemphigus, bullous pemphigoid, papular acrodermatitis, erythromelalgia, and vasculitis following influenza vaccinations [24]. However, there are still only a few reports (one case report and one observational clinical study) in the literature describing the new onset and/or exacerbation of psoriasis following influenza vaccination [16, 17]. Shin et al. recently described a 26-year-old woman with multiple erythematous scaly macules scattered on the extremities and trunk compatible with psoriasis, who had been injected with an inactivated split-virus influenza A/H1N1 vaccine without adjuvant (Green Flu-S, Green Corp.) [16]. So far, only one available clinical study has been carried out into the triggering effect of influenza vaccination on psoriasis. In that study, Sbidian et al. evaluated psoriasis patients who had been vaccinated against influenza and had exhibited induction or exacerbation of psoriasis. They sent a declaration request questionnaire once by e-mail to nearly 3,000 French dermatologists through the institutional channels to request reporting any case who describes psoriasis onset or flare after H1N1/seasonal vaccination. They received feedback from 6 dermatologists concerning 10 patients presenting with a psoriasis of new onset (*n* = 7) or with a worsening of previously diagnosed psoriasis (*n* = 3) within 3 months following the 2009 monovalent H1N1/seasonal vaccination. Among them, six patients with the first episode of psoriasis had exhibited the guttate/plaque mixed clinical phenotype, while the remaining patient had displayed a plaque type with a median onset of 8 days after vaccination (range 6–74 days). Even though data for these patients is available, the date could not provide a reliable estimation of the rate of vaccination-related reaction because of the uncertainties concerning the actual number of psoriatic patients undergoing vaccination, an underestimation of the incidence due to underreporting, and underdiagnosis. Consequently, the authors claim that even if it is not a very strong effect, influenza vaccination is associated with psoriasis flare [17].

Our study has a number of limitations including the lack of a control group and follow-up evaluations which could prove causal correlation between vaccine and clinical manifestation. Despite these potential limitations, our observations may partially support the apparent association between influenza vaccination and the development of psoriasis. The fact that no other provoking factors were found in our patients promotes this relationship.

Although staphylococcal, streptococcal, measles, and varicella vaccines have also been applied previously in the treatment of psoriasis, an early report described two cases of psoriasis induced by BCG vaccination and influenza vaccination under the name of “psoriasis vaccinalis” [10, 20]. Subsequently, extremely rare cases of psoriasis, psoriasis-like guttate eruptions, and psoriatic arthropathy have been reported following BCG vaccination and a case of psoriasis was reported, as triggered by the tetanus-diphtheria vaccination [11–14]. Additionally, a case-control study reported rubella vaccination as a risk factor for psoriatic arthropathy [15]. The etiological relationship between psoriasis and the vaccination remains uncertain. It was claimed that the vaccination may trigger an exacerbation of psoriatic skin lesions itself; however, these views also concur with the well-known Koebner phenomenon that occurs in psoriasis, that is, the development of new plaques at the locations of skin injury [1]. Researchers also indicated that the BCG and tetanus-diphtheria vaccines, due to the mycobacterial heat shock proteins in the case of the BCG and diphtheria toxoid in the case of the tetanus-diphtheria vaccines, induce IL-6 production, which, in turn, promotes the development of Th17 cells [12–14]. Previous studies had also demonstrated a significant increase in IL-6 values after the administration of the influenza vaccination [25, 26]. In the study of the influenza vaccine in a murine model, the cytokine profile demonstrated a robust cellular immune response with enhanced Th1 and Th17 immunity that provided balanced immunity against both intracellular and extracellular forms of the virus [27]. Consistent with these findings, it could be speculated that an immunological reaction to the influenza vaccination may mediate the IL-6 production and generation of IL-22-producing Th17 cells which are a key player in the development of characteristic epidermal changes of psoriasis [9, 16, 17]. These mechanisms are also probably responsible for the activation of the immune system and provocation of psoriasis in our cases. However, positive rechallenges may provide some support for a causal relationship between a vaccine and an adverse event and represent a reasonable basis for further assessment [17].

## 4. Conclusion

Consequently, our data suggests that the H1N1 influenza vaccines, which were used in the 2009-2010 season, have the potential to trigger development of psoriasis. Therefore, it is important not only to know the protective potential of influenza vaccines but also to understand their potential to provoke psoriasis.

However, as previously suggested, although the administration of the influenza vaccine has been associated with psoriasis in some patients, their very low incidence and mild clinical course, combined with the general lack of high level of evidence, do not warrant an abandonment of the immunization practice considering the favorable cost-effectiveness ratio of the vaccine use. Therefore, we recommend the follow-up of such individuals and suggest further large-sized, controlled, and well-constructed clinical research studies going forward, which may confirm this relationship.
